# A multifaceted clinical portrait of Ebstein’s anomaly: a case series

**DOI:** 10.1093/ehjcr/ytaf424

**Published:** 2025-09-02

**Authors:** Panayotis K Vlachakis, Eva Nyktari, George Oikonomou, Anastasios Apostolos, Skevos Sideris, Konstantinos Toutouzas, Costas Tsioufis, Maria Drakopoulou

**Affiliations:** Adult Congenital Heart Centre and Centre for Pulmonary Hypertension, First Cardiology Department, Hippokration General Hospital, Athens Medical School, National and Kapodistrian University of Athens, 114 Vasilissis Sophias, Athens 11527, Greece; CMR Unit, Onassis Cardiac Surgery Center, 356 Andrea Syggrou Avenue, Kallithea 17674, Greece; Adult Congenital Heart Centre and Centre for Pulmonary Hypertension, First Cardiology Department, Hippokration General Hospital, Athens Medical School, National and Kapodistrian University of Athens, 114 Vasilissis Sophias, Athens 11527, Greece; Adult Congenital Heart Centre and Centre for Pulmonary Hypertension, First Cardiology Department, Hippokration General Hospital, Athens Medical School, National and Kapodistrian University of Athens, 114 Vasilissis Sophias, Athens 11527, Greece; Department of Cardiology, Hippokration General Hospital, 114 Vasilissis Sophias, Athens 11527, Greece; Adult Congenital Heart Centre and Centre for Pulmonary Hypertension, First Cardiology Department, Hippokration General Hospital, Athens Medical School, National and Kapodistrian University of Athens, 114 Vasilissis Sophias, Athens 11527, Greece; Adult Congenital Heart Centre and Centre for Pulmonary Hypertension, First Cardiology Department, Hippokration General Hospital, Athens Medical School, National and Kapodistrian University of Athens, 114 Vasilissis Sophias, Athens 11527, Greece; Adult Congenital Heart Centre and Centre for Pulmonary Hypertension, First Cardiology Department, Hippokration General Hospital, Athens Medical School, National and Kapodistrian University of Athens, 114 Vasilissis Sophias, Athens 11527, Greece

**Keywords:** Ebstein’s anomaly, Tricuspid regurgitation, Right ventricle, Valve-in-valve, Accessory pathway, Case report

## Abstract

**Background:**

It was in 1866 when Wilhelm Ebstein first described a rare case of a 19-year-old cyanotic patient with tricuspid valve regurgitation caused by a congenital malformation. Since then, Ebstein’s anomaly (EA) has been recognized as a condition that exhibits a diverse clinical course with a formidable challenge for clinicians in terms of diagnosis, management, and prognosis.

**Case summary:**

We present a case series of four patients with EA, shedding light on the varied clinical manifestations, treatment approaches, and outcomes that underscore the multifaceted nature of this condition.

**Discussion:**

The management of EA requires a comprehensive and individualized approach in specialized centres. The cardiologists responsible for the long-term care of these patients are faced with various challenging ‘dilemmas’ in the clinical course of the disease at different stages. The evolving landscape of cardiology offers a promising avenue for improving the outcomes and quality of life of patients with EA.

Learning points

- In Ebstein’s anomaly, the spectrum of the disease is diverse, with varied clinical manifestations, treatment approaches, and outcomes that underscore the multifaceted nature of this condition. Due to various challenging ‘dilemmas’ in the clinical course of these patients, multi-disciplinary approach is needed to facilitate tailored patient care and optimize long-term prognosis.
- Timely surgical intervention with a preference to tricuspid valve repair over replacement therapy is critical in Ebstein’s anomaly, as delaying surgery is associated with unfortunate outcomes.
- Transcatheter valve-in-valve implantation in patients with Ebstein’s anomaly and malfunctioning bioprosthetic valve in the tricuspid position requires detailed anatomical planning and seems an attractive alternative to repeat surgery in high-risk or multi-operated patients.
- Accessory pathways are common in Ebstein’s anomaly and carry a high arrhythmic risk, necessitating early electrophysiological evaluation and consideration of catheter ablation for long-term rhythm control.

## Introduction

It was in 1866 when Wilhelm Ebstein first described a rare case of a 19-year-old cyanotic patient with tricuspid valve regurgitation caused by a congenital malformation.^1^ Since then, Ebstein’s anomaly (EA) has been recognized as a condition that exhibits a diverse clinical course with a formidable challenge for clinicians in terms of diagnosis, management, and prognosis. Herein, we present a case series of four patients with EA, shedding light on the varied clinical manifestations, treatment approaches, and outcomes that underscore the multifaceted nature of this condition. The series emphasizes the need for a nuanced understanding of the disease to facilitate tailored patient care and optimize long-term prognosis.

## Summary figure

**Figure:**
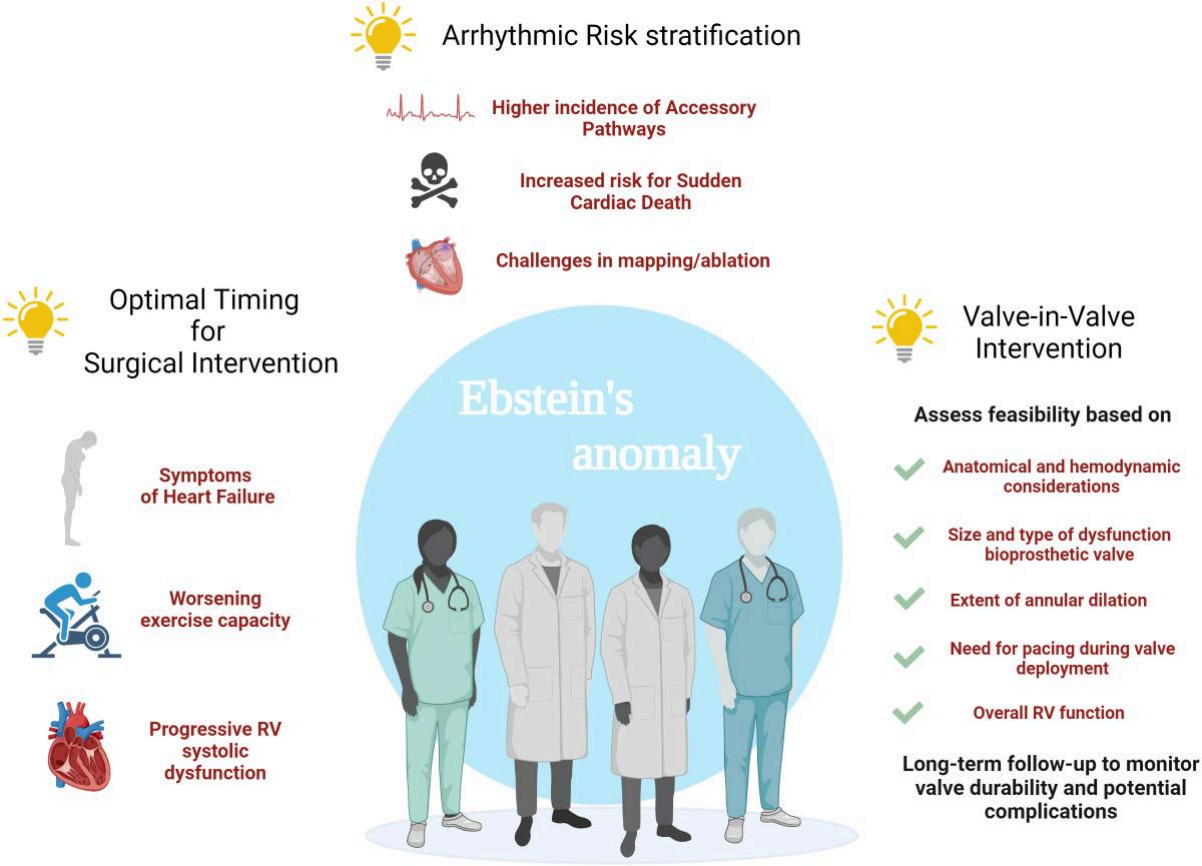
The management of EA requires a comprehensive and individualized approach in specialized centres. The cardiologists responsible for the long-term care of these patients are faced with various challenging ‘dilemmas’ in the clinical course of the disease at different stages.

## Clinical cases

### Case 1

A 67-year-old woman with EA not diagnosed until late adulthood was referred to our hospital due to mild exercise restriction of recent onset. The patient has been in a good functional class until now without the need for surgical correction.

**Figure 1:**
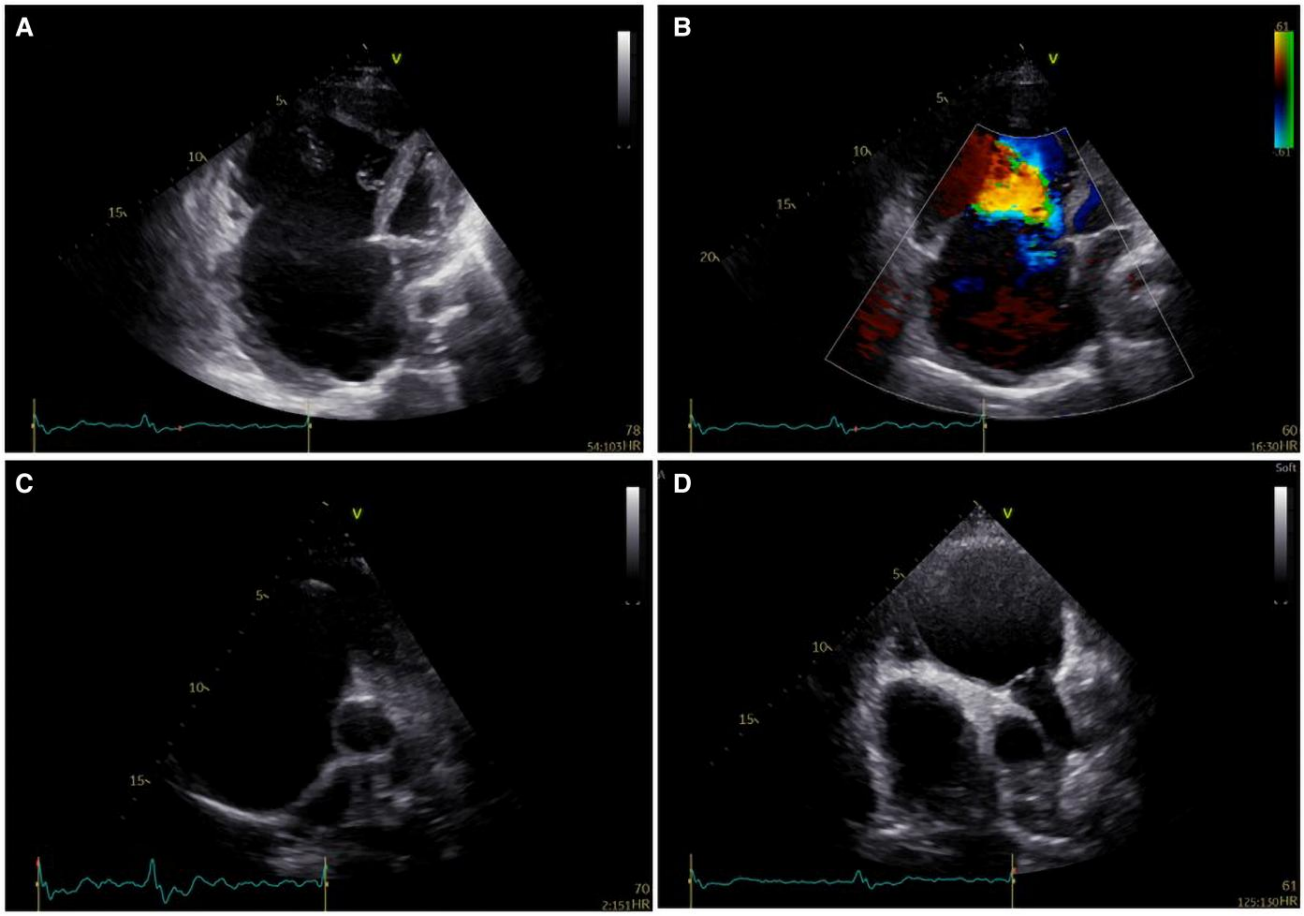
Apical four-chamber view by 2D echocardiography (*A*) and Doppler imaging (*B*) with a focus on right ventricular inflow showing extreme apical displacement of the septal and posterior leaflets whereas the anterior leaflet appeared sail-like and tethered at its mid and distal position with complete malcoaptation of the tricuspid valve leaflets with torrential regurgitation. The image illustrates a giant RA and severe dilation of the functional apical portion of the RV. The LV shows a small cavity with preserved systolic function, low cardiac output, and leftward displacement due to severe dilation of the right heart. Short axis view by 2D echocardiography with a focus on RV inflow (*C*) and RV outflow (*D*) showing severe RV dilation. LV, left ventricle; RA, right atrium; RV, right ventricle.

On physical examination, the patient was stable with no signs of right heart failure. There was mild cyanosis with an O_2_ saturation of ∼85% on air. On auscultation, there was a systolic murmur and increased jugular venous pressure (JVP) with no prominent V wave. The electrocardiogram (ECG) revealed sinus rhythm, with prominent P waves, first degree atrioventricular block, and right bundle branch block (QRS duration ∼155 ms). Blood tests showed secondary erythrocytosis (Hct ∼60%) due to chronic hypoxia and increased natriuretic peptides but were otherwise unremarkable. Echocardiography and cardiac magnetic resonance imaging showed extreme apical displacement of the septal and posterior leaflets whereas the anterior leaflet appeared sail-like and tethered at its mid and distal position (*Figure 1*, Supplementary material online, *Figure S1*, Supplementary material online, *Video S1*). There was no interatrial communication. Based on cardiovascular magnetic resonance (CMR) measurements, there was significant atrialization of the right ventricular (RV) inflow resulting in a giant right atrium and torrential tricuspid regurgitation due to malcoaptation of the leaflets (see Supplementary material online, *Table S1*). The RV overall presented with severe dilation at its apical and functional component with impaired systolic function in the presence of significant volume overload (see Supplementary material online, *Figure S2*). The left ventricle was small in size, compromised and displaced to the left (severe RV dilation) with preserved systolic function and low cardiac output. During early diastole the left ventricle was D-shaped due to the significant RV volume overload. Left and right heart cardiac catheterization showed no significant coronary artery disease and low pressure in pulmonary vasculature. The patient was discussed at the multi-disciplinary team (MDT) meeting for contemporary tricuspid repair techniques and/or bidirectional cavopulmonary anastomosis. Whilst surgical intervention and staged procedures were discussed, as the patient had only slight limitations on ordinary physical activity, both the team and patient favoured conservative therapy (low dose of diuretics) and watchful waiting.

### Case 2

A 14-year-old asymptomatic patient with a congenital background of EA associated with a perimembranous ventricular septal defect (VSD) (spontaneously closed during early childhood), was seen at the transition from paediatrics to adult care of our academic centre.

**Figure 2:**
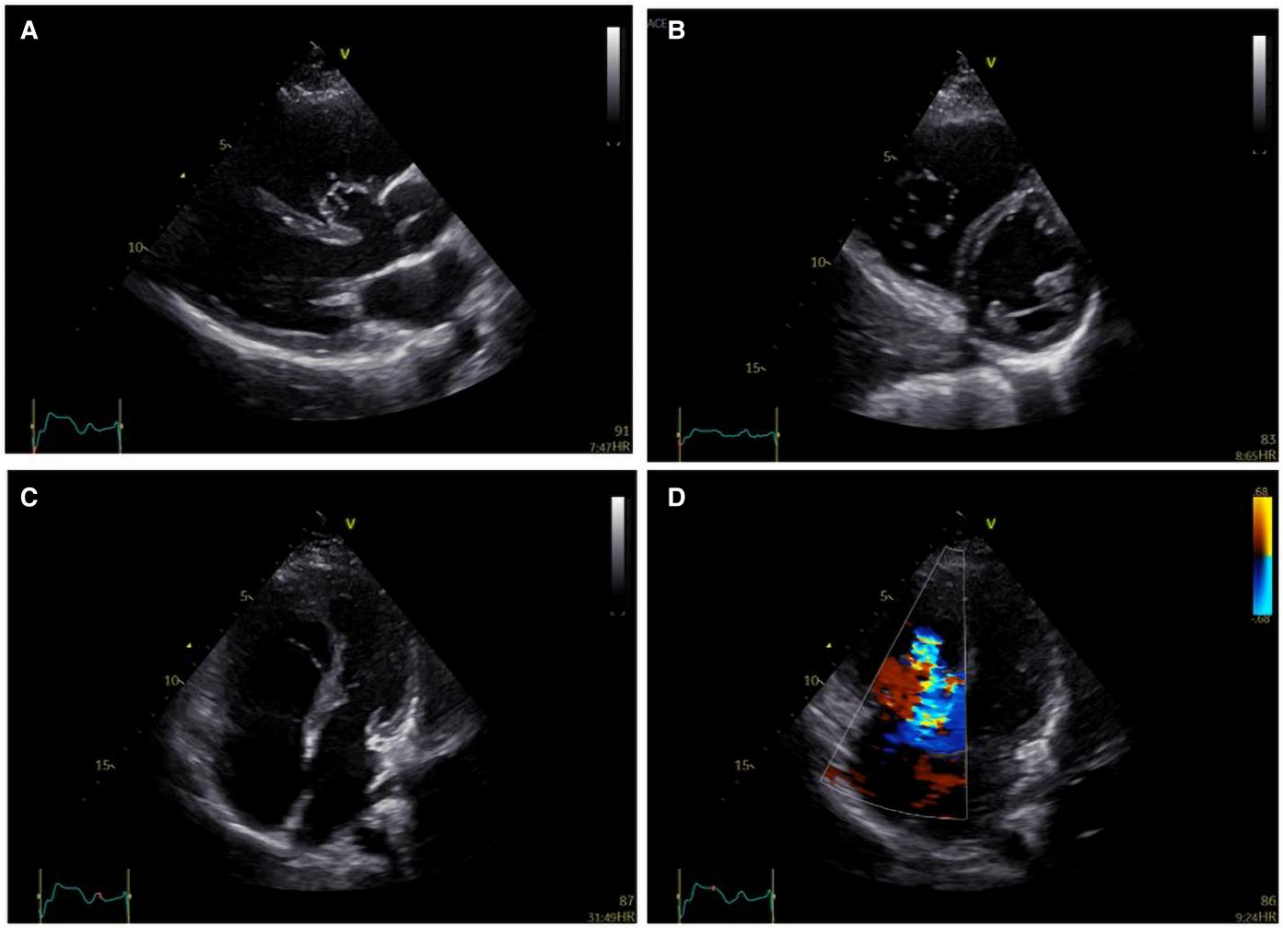
(*A*) Parasternal long axis view by transthoracic echocardiography showing the spontaneous closure of a VSD by formation of an aneurysm of the membranous septum. The RV is dilated with preserved systolic function. (*B*) Parasternal short axis view by transthoracic echocardiography at the level of papillary muscles showing the dilated RV and the short axis of tricuspid valve leaflets that are displaced towards the apex. (*C*) Apical four-chamber view by transthoracic echocardiography showing marked apical displacement of the septal leaflet (∼28 mm, 17 mm/m^2^) and an elongated ‘sail-like’ anterior leaflet. (*D*) Apical four-chamber view by transthoracic echocardiography with Doppler imaging showing significant tricuspid regurgitation. RV, right ventricle; VSD, ventricular septal defect.

**Figure 3:**
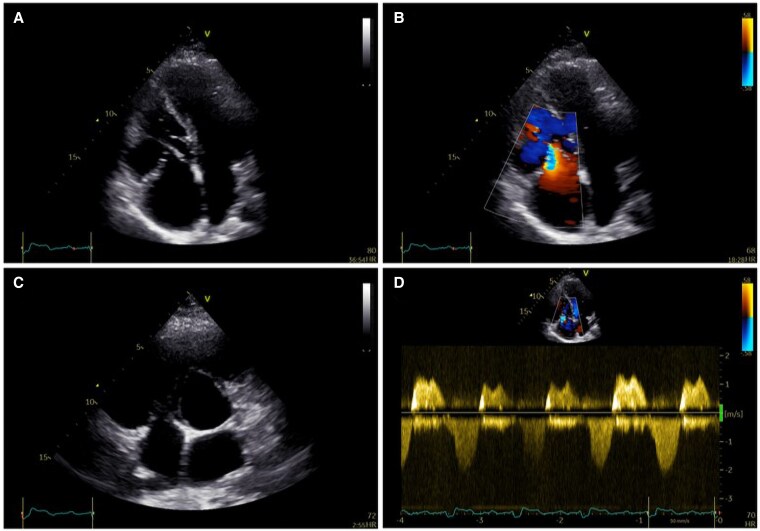
(*A*, *B*) Apical four-chamber view showing a well-functioning tricuspid valve after repair by 2D transthoracic echocardiography (*A*) and mild to moderate tricuspid regurgitation with Doppler imaging (*B*). (*C*) Short axis view with a focus on tricuspid valve showing good motion of tricuspid valve leaflets and the presence of the spontaneously closed VSD. (*D*) Continuous wave Doppler by transthoracic echocardiography at the tricuspid valve after repair depicting transvalvular mean gradient of ∼3 mmHg at 70 b.p.m. in the presence of mild to moderate regurgitation. RV, right ventricle; VSD, ventricular septal defect.

The patient was in a good functional class. On examination, the heart rate was regular at ∼80 b.p.m., the blood pressure 110/75 mmHg, and the JVP was normal. On auscultation, first and second heart sounds were audible along with a systolic murmur. ECG showed sinus rhythm with first degree atrioventricular block and right bundle branch block (QRS duration ∼145 ms). Lab tests were unremarkable, and brain natriuretic peptides were at the upper normal limit adjusted for age. During cardiopulmonary exercise test there was no desaturation. However, imaging by transthoracic echocardiography (*Figure 2*, Supplementary material online, *Video S2*) and CMR (see Supplementary material online, *Figure S2*, Supplementary material online, *Table S1*), showed severe tricuspid regurgitation due to marked apical displacement of the septal leaflet and an elongated ‘sail-like’ anterior leaflet. Based on CMR measurements, the inflow component of the RV was atrialized, while the remaining functional component was severely dilated with impaired systolic function (47%) in the presence of significant volume overload (see Supplementary material online, *Figure S2*). Although there was no objective deterioration of exercise capacity, the MDT meeting decided for surgical correction of EA in this patient due to the severe tricuspid regurgitation and significant RV dilation with impaired systolic function in the presence of significant volume overload on CMR study. The patient underwent successful surgical repair using the Carpentier’s technique including extensive mobilization and relocation of tricuspid leaflets to the normal tricuspid annulus, longitudinal plication of the atrialized chamber with reconstruction of the RV ventricle and tricuspid annuloplasty.^2,3^ On recent follow-up, the patient remains asymptomatic while not on medical therapy. Transthoracic echocardiography showed a mildly elevated transvalvular gradient in the presence of mild to moderate tricuspid regurgitation (*Figure 3*).

### Case 3

A 53-year-old patient, with a cardiac background of EA associated with patent ductus arteriosus and corrective surgery ∼11 years ago, was admitted in our hospital with progressive dyspnoea in poor functional class (NYHA class III). At the time of surgery, the patient received a bioprosthesis at the tricuspid position (31 mm Carpentier-Edwards Perimount Magna, Edwards Lifesciences, Irvine, CA) and underwent closure of the patent ductus arteriosus. After surgery, due to complete heart block the patient received an epicardial pacemaker.

On admission, the patient was stable with signs of right heart failure as manifested by oedema and elevated JVP, requiring moderate-to-high doses of diuretics. On auscultation, a mid-late diastolic rumble was audible. ECG showed sinus rhythm with right bundle branch block (QRS duration ∼145 ms). Natriuretic peptides were mildly elevated (NT-proBNP 1679 pg/mL), whereas other blood tests were unremarkable.

**Figure 4:**
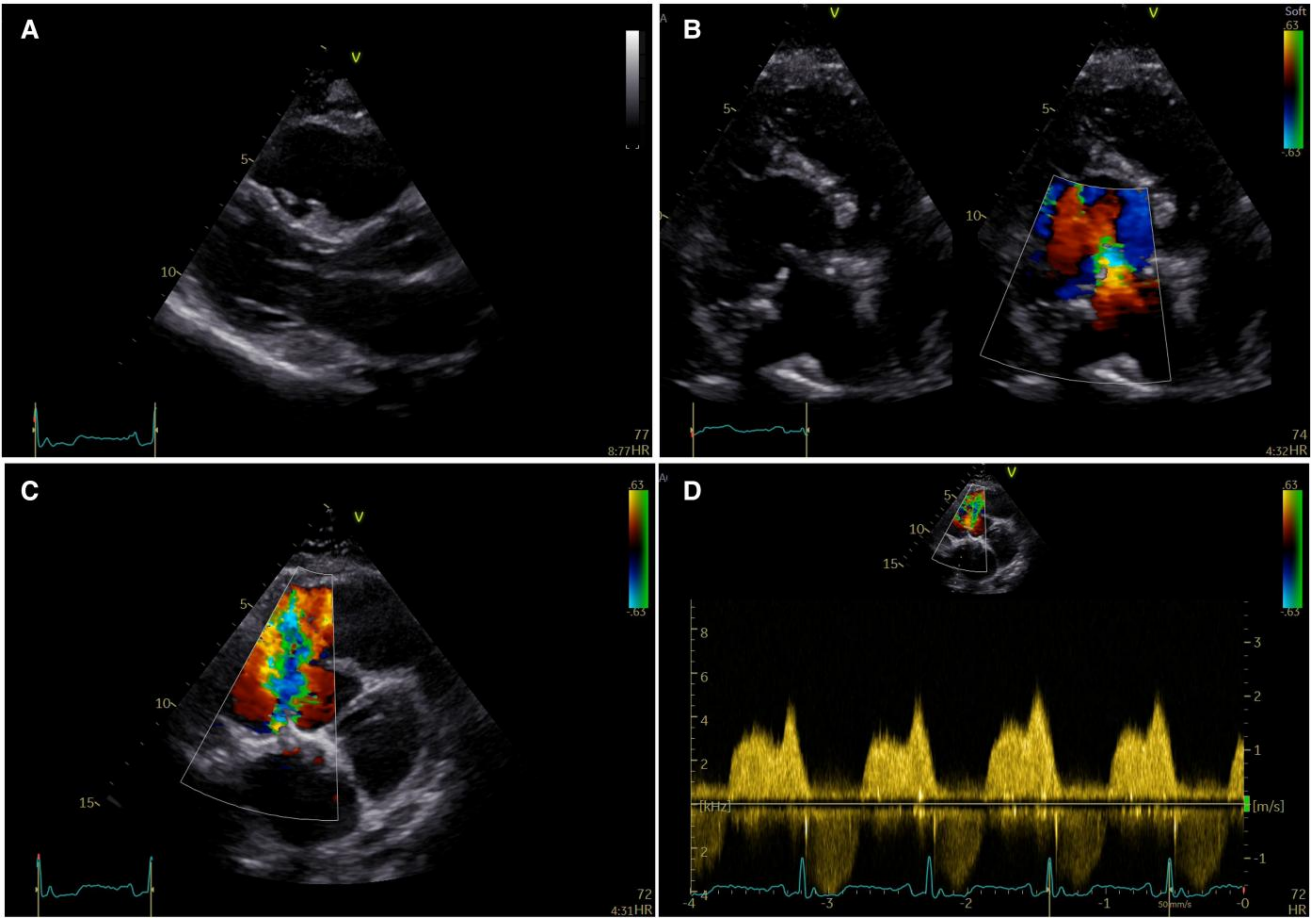
(*A*) Parasternal long axis view by transthoracic echocardiography showing dilated RV with impaired systolic function. (*B*) Apical four-chamber view by 2D transthoracic echocardiography with Doppler imaging with a focus on bioprosthesis at the tricuspid position showing increased flow velocity across the valve. (*C*) Apical four-chamber view with a focus on right ventricular inflow by 2D echocardiography and Doppler imaging showing thickened bioprosthesis valve leaflets with reduced mobility, significant stenosis, and at least moderate regurgitation. (*D*) Continuous wave Doppler imaging showing significant bioprosthesis valve stenosis (mean tricuspid gradient ∼10.9 ± 3.9 mmHg at ∼75 b.p.m.) and at least moderate regurgitation. RV, right ventricle; VSD, ventricular septal defect.

**Figure 5:**
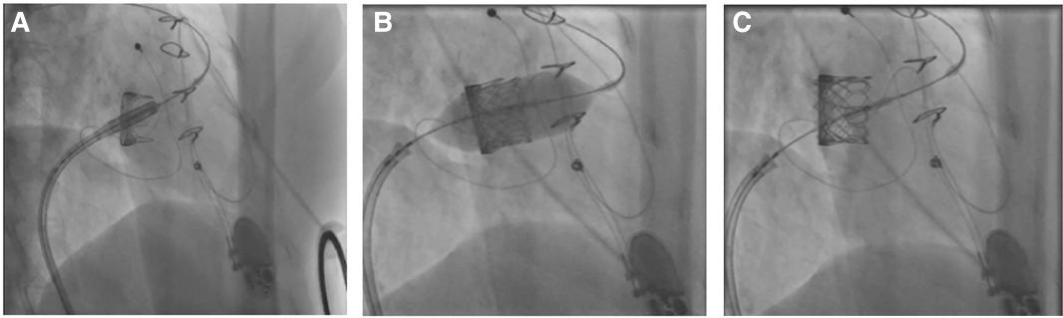
Fluoroscopy images depicting transcatheter valve-in-valve procedure (step-by-step) in the tricuspid position. Sequence of the release of prosthesis. Slow balloon inflation (*A*), inflation to full volume (*B*), final transcatheter valve position (*C*). The transcatheter valve is *in situ* and well-seated.

**Figure 6:**
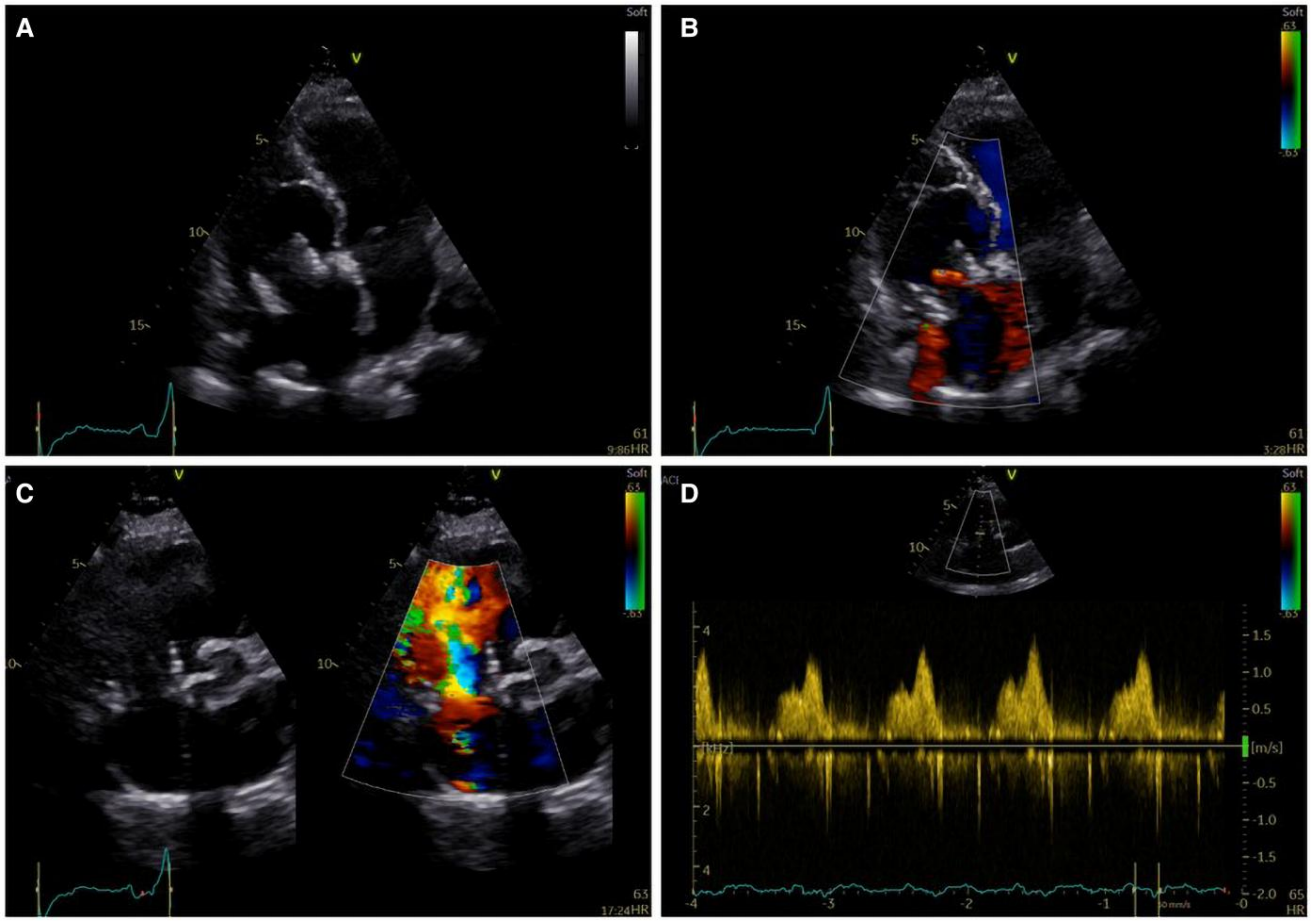
Apical four-chamber view by 2D echocardiography (*A*) and Doppler imaging (*B*) with a focus on RV inflow showing good function of the transcatheter valve in the tricuspid position. Short axis view by 2D echocardiography and Doppler imaging (*C*) with a focus on RV inflow showing laminar flow in the tricuspid valve prosthesis with no para-valvular or transvalvular regurgitation. (*D*) Continuous wave Doppler imaging showing good function of transcatheter valve in the tricuspid position with a mean gradient ∼1 mmHg at 65 b.p.m. RV, right ventricle.

In-hospital transthoracic and transoesophageal echocardiography showed preserved biventricular function. The bioprosthesis at the tricuspid position showed significant degeneration with restricted leaflet motion restriction due to severe calcification. There was bioprosthesis dysfunction with severe stenosis and at least moderate regurgitation (*Figure 4*, Supplementary material online, *Video S3*). The indication and therapeutic approach were discussed at the MDT meeting. Taking into consideration the limited evidence for isolated tricuspid valve reoperations and the feasibility of transcatheter replacement therapies for failed bioprosthesis in the tricuspid position, there was a consensus for valve-in-valve procedure.^4^ The patient underwent further screening and imaging with cardiac computer tomography (CT): The valve annulus area was ∼860 mm^2^ and the perimeter was ∼98 mm. Based on manufacturer information, the prosthetic valve had an ∼31 mm internal diameter (ID) and a 29 m true ID. Thus, a 29 mm balloon-expandable valve (Myval, Meril) was selected for this patient. The procedure was performed under deep sedation and the valve was slowly deployed and anchored in the previous calcified leaflets and ring (*Figure 5*). Following valve implantation, the patient showed an immediate improvement in haemodynamics, maintaining circulatory stability. Dismissal transthoracic echocardiography showed a well-seated transcatheter valve with a transvalvular mean gradient of ∼2 mmHg at 65 b.p.m. and no regurgitation. The favourable result is retained at 7-year follow-up (*Figure 6*) while the patient is still on single antiplatelet therapy, antihypertensive treatment (angiotensin-converting-enzyme inhibitors, ACE inhibitors) and low dose of diuretics.

### Case 4

**Figure 7:**
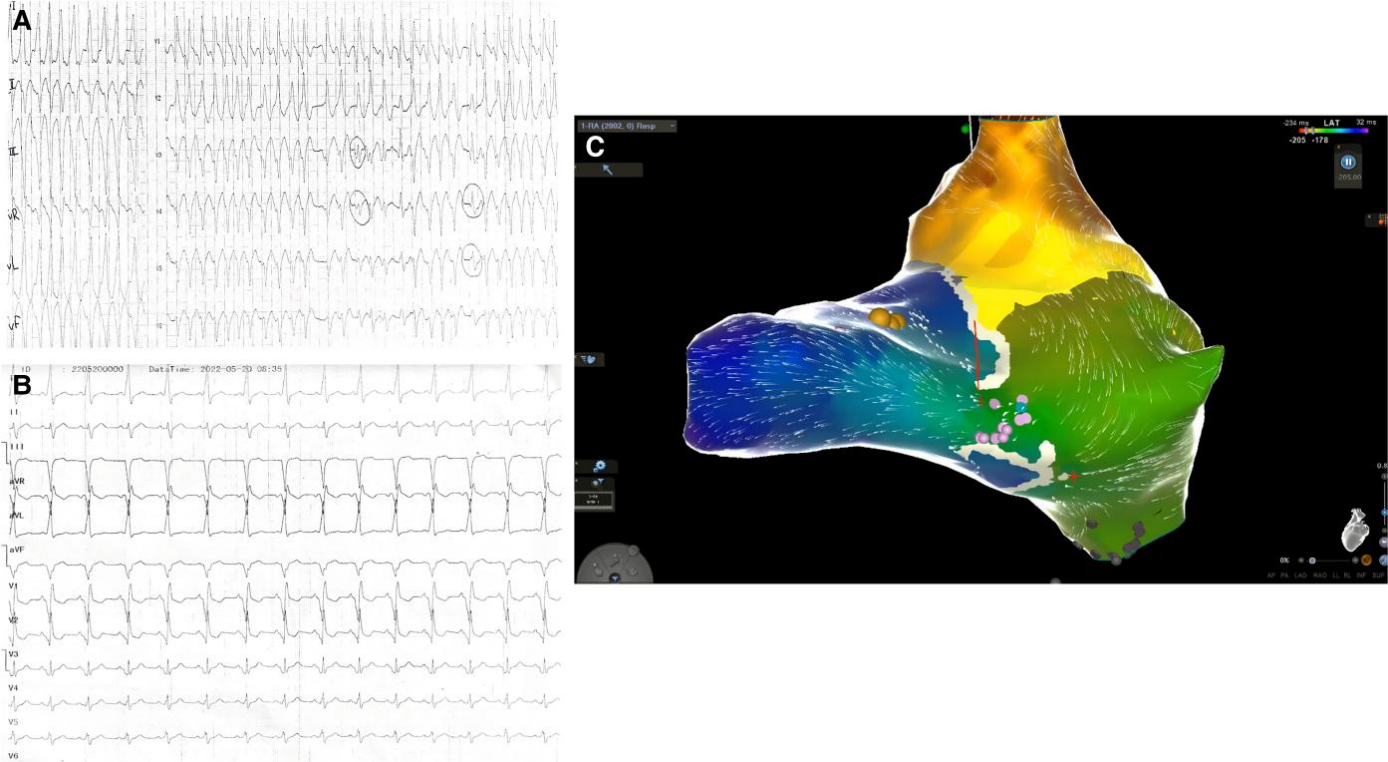
(*A*) Fast, irregular broad complex tachycardia, typically indicative of pre-excited atrial fibrillation. (*B*) Post-cardioversion ECG showing negative delta waves in leads II, aVF, and V1 and positive in lead I. (*C*) 3D-electroanatomical activation mapping of the RA of the patient during sinus rhythm. The vertical white lines represent the tricuspid valve plane, which is electrically inert, the yellow dots stand for the anatomical area of the His bundle while the purple dots depict the spots where AP potentials were recorded with the Pentarray catheter (Carto 3 System, Biosense Webster, Johnson and Johnson) as well as the propagation wavefront in the gigantic RA and through the AP to the RV. 3D, three-dimensional; AP, accessory pathway; ECG, electrocardiogram; RF, radiofrequency; RA, right atrium; RV, right ventricle.

A 22-year-old male with uncorrected EA presented to our emergency department complaining of dizziness and heart palpitations of recent onset. The electrocardiography showed pre-excited atrial fibrillation, and due to haemodynamic instability, the patient underwent successful direct current cardioversion (*Figure 7*). In the ECG post-cardioversion, pre-excitation was prominent with negative delta waves in leads V1, III, aVF, and positive delta wave in lead V3. After cardioversion the patient remained stable with no signs of heart failure. On auscultation, there was an audible systolic murmur of grade 3. Echocardiography identified the septal and posterior leaflets of the tricuspid valve as displaced towards RV apex, with a dilated atrialized portion of the RV but with preserved systolic function (see Supplementary material online, *Figure S3*, Supplementary material online, *Video S4*). An electrophysiology study revealed the presence of a right nodo-hisian accessory pathway (AP), prompting the performance of successful catheter ablation (*Figure 7*). On recent follow-up, the patient remains free of arrhythmia under no medical therapy.

## Discussion

The incidence of EA in the general population has been approximated to range for 1.2–5 per 100 000 live births.^5^ This distinct clinical entity demonstrates a spectrum of severity, encompassing infants who do not survive early childhood, asymptomatic adults incidentally diagnosed in their sixth and seventh decades of life, and various degrees of severity distributed across the spectrum.^6^ Multimodality imaging is crucial to acquire comprehensive insights into the intricate anatomy of right atrium and RV, detecting coexisting defects, assessing tricuspid valve function and guiding surgical interventions.^7^

The management concerning the anatomical background involves close monitoring, pharmacological therapy and surgical or catheter-based procedures. In line with current guidelines, adults with EA and significant tricuspid regurgitation undergo surgical repair or reoperation, particularly in the presence of heart failure symptoms, objective evidence of worsening exercise capacity, and/or progressive RV systolic dysfunction.^7–10^ Patients with minimal symptoms presenting in late adulthood, however, may warrant patient-directed care, creating the dilemma of whether the risk outweighs the benefit of surgery. In asymptomatic patients, identifying the optimal timing for surgical intervention is of paramount importance, as data highlight that postponing surgery until the onset of heart failure or RV systolic dysfunction results in less favourable outcomes.^11^ Although operative mortality is exceptionally low in patients beyond infancy, whether early diagnosis and surgical intervention translate into better clinical outcomes is a critical question to be answered in the coming decades.^12^ Last but foremost, once surgical management is indicated, variation of Ebstein valve morphology affects surgical strategy. Among other surgical strategies cone procedure, pioneered by Jose da Silva, MD, is the standard treatment for patients with EA.^13^ Still, optimal case selection and patient-specific planning based on patients’ anatomical criteria appear to be crucial factors for success. Hopefully, large multicentre studies will allow us to address these unanswered questions.

Patients with EA and a history of prior surgical valve replacement represents a group known for its challenging management, with limited options due to the higher mortality risk of a redo tricuspid valve replacement and repair.^14,15^ It is essential to assess the anatomical and haemodynamic considerations specific to each patient to determine the feasibility and appropriateness of a valve-in-valve procedure that seems a viable and effective treatment option with promising outcomes. Factors such as the size and type of the dysfunctional bioprosthetic valve, the extent of annular dilation, pacing need for valve deployment, and the overall RV function should be thoroughly evaluated to ensure optimal procedural outcomes.^16^ Long-term follow-up is necessary to monitor the durability and performance of the transcatheter-implanted valve and to assess any potential complications that may arise. The introduction, however, of tricuspid valve-in-valve procedure now offers a therapeutic option for patients with once prohibitive or exceedingly high reoperation risk.

Besides the anatomical background, patients with EA are known to have an increased incidence of AP, ∼10–30%, which is attributed to the abnormal anatomy of the tricuspid valve and right atrium.^17–19^ The presence of one or more APs may lead to a variety of arrhythmias, including high-risk pathways that exhibit an increased risk of sudden cardiac death, largely associated with ventricular fibrillation resulting from rapidly conducting atrial fibrillation. The complexity of the anatomical considerations contributes to the persistent challenges in mapping and catheter ablation of these APs.^18^ Still, knowledge of the anatomy and risk of AP in Ebstein’s can direct successful ablation in these challenging patients who are at high risk of sudden cardiac death from fast ventricular conduction AF and in whom medical management is rarely successful and appropriate.

## Conclusion

The management of EA requires a comprehensive and individualized approach in specialized centres. The cardiologists in charge for the long-term care of these patients are faced with various challenging ‘dilemmas’ in the clinical course of the disease at different stages. The evolving landscape of cardiology offers a promising avenue for improving the outcomes and quality of life of patients with EA.

## Lead author biography


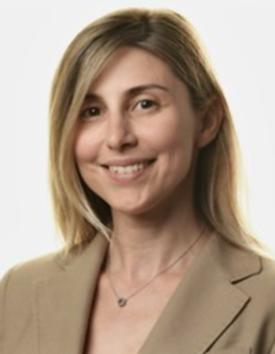


Dr Maria Drakopoulou is a consultant cardiologist and the lead of the adult congenital heart disease (ACHD) and pulmonary hypertension (PH) unit in the First Department of Cardiology of Athens Medical University. After completion of her residency training in Athens, she undertook a fellowship in ACHD and advanced cardiac imaging in Royal Brompton Hospital, UK. She has a keen clinical and academic interest in the diagnosis and management of patients with structural and/or congenital heart disease with a view to enhance patient health outcomes and quality of life.

## Supplementary Material

ytaf424_Supplementary_Data

## Data Availability

All data are incorporated into the article and its online Supplementary material.
