# Supplementary material for: A multifaceted clinical portrait of Ebstein’s anomaly: a case series
Source: Eur Heart J Case Rep. 2025 Sep 2;9(9):ytaf424. doi: 10.1093/ehjcr/ytaf424 (PMC12461249; doi:10.1093/ehjcr/ytaf424)
Supplement: ytaf424_Supplementary_Data [file ytaf424_supplementary_data.zip › Table.docx]

**Supplementary Table 1.**

|  |  | **Case 1** | **Case 2** |
| --- | --- | --- | --- |
| **Right Ventricle** | Indexed EDV* | 309 ml/m^2^ | 154 ml/m^2^ |
|  | Indexed ESV* | 145 ml/m^2^ | 68 ml/m^2^ |
|  | EF | 52 % | 47 % |
| **Left Ventricle** | Indexed EDV* | 42 ml/m^2^ | 61 ml/m^2^ |
|  | Indexed ESV* | 13 ml/m^2^ | 23 ml/m^2^ |
|  | EF | 69 % | 63 % |
| **Tricuspid Valve** | Vena Contracta | 15mm | 6mm |
|  | Regurgitant Volume | 240ml | 76ml |
|  | Regurgitant Fraction | 83% | 56% |
| **Right atrium indexed area** | | 44cm^2^/m^2^ | 21 cm^2^/m^2^ |

*EDV: end-diastolic volume, ESV: end-systolic volume
