# Supplementary figures and images for: A multifaceted clinical portrait of Ebstein’s anomaly: a case series
Source: Eur Heart J Case Rep. 2025 Sep 2;9(9):ytaf424. doi: 10.1093/ehjcr/ytaf424 (PMC12461249; doi:10.1093/ehjcr/ytaf424)

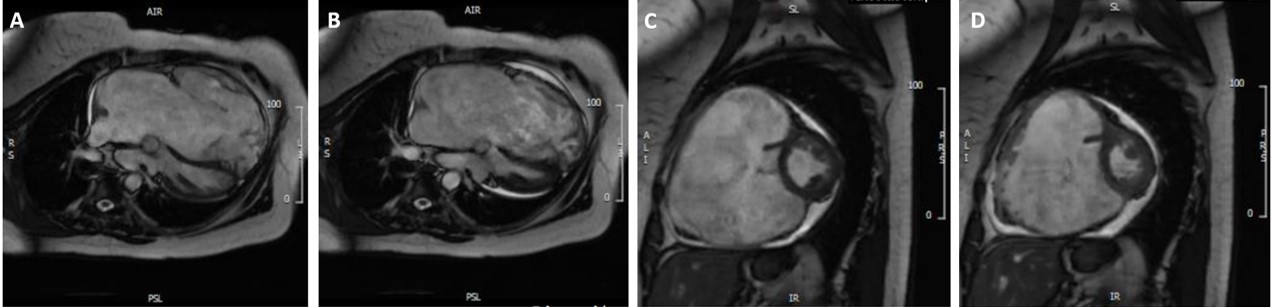

Supplement: ytaf424_Supplementary_Data [file ytaf424_supplementary_data.zip › Figure 1 supplementary revision 5.jpg]

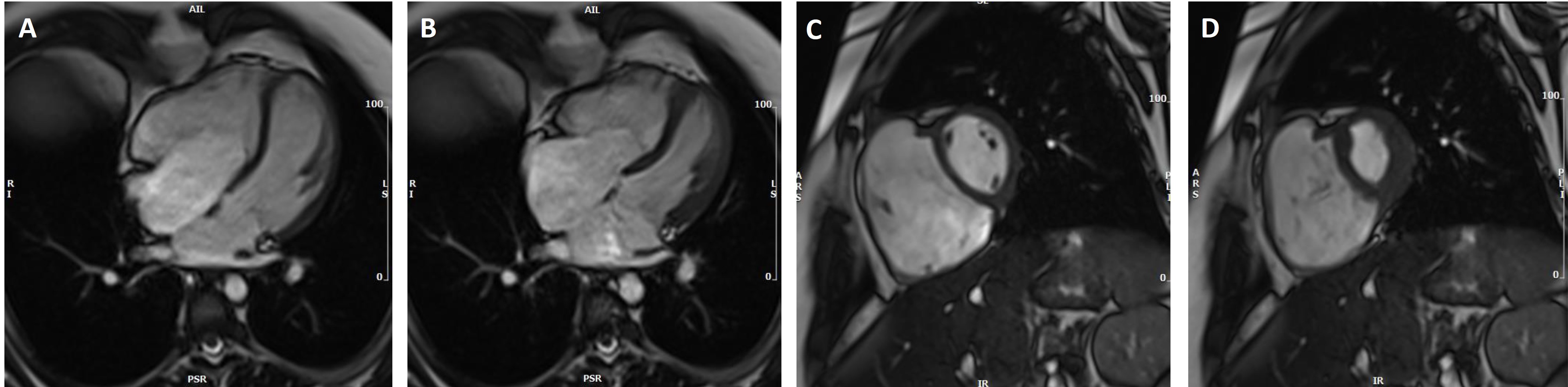

Supplement: ytaf424_Supplementary_Data [file ytaf424_supplementary_data.zip › Figure 2 supplementary revision 5.jpg]

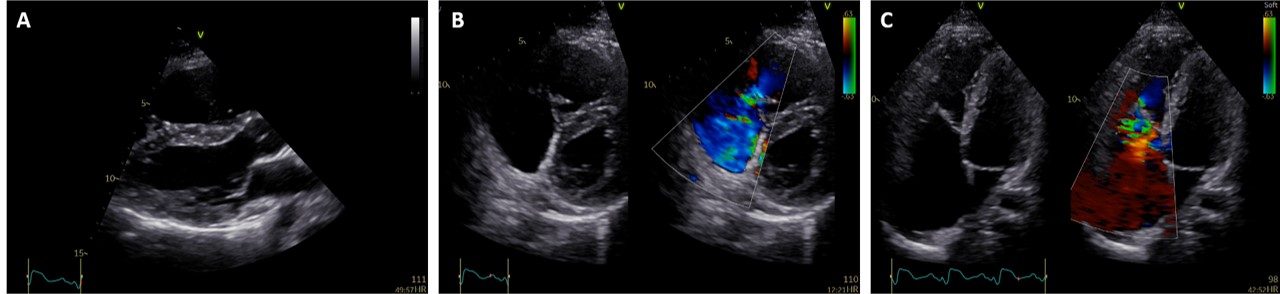

Supplement: ytaf424_Supplementary_Data [file ytaf424_supplementary_data.zip › Figure 3 supplementary revision 5.jpg]
